# ﻿Two novel hyphomycetes associated with ferns from China

**DOI:** 10.3897/mycokeys.113.137678

**Published:** 2025-01-31

**Authors:** Jing-Yi Zhang, Kevin D. Hyde, Li-Juan Zhang, Song Bai, Dan-Feng Bao, Fatimah Al-Otibi, Yong-Zhong Lu

**Affiliations:** 1 School of Food and Pharmaceutical Engineering, Guizhou Institute of Technology, Guiyang 550003, China; 2 Center of Excellence in Fungal Research, Mae Fah Luang University, Chiang Rai 57100, Thailand; 3 School of Science, Mae Fah Luang University, Chiang Rai 57100, Thailand; 4 Department of Botany and Microbiology, College of Science, King Saud University, P.O. Box 22452, Riyadh 11495, Saudi Arabia; 5 Guizhou Industry Polytechnic College, Guiyang 550008, China; 6 Engineering and Research Center for Southwest Bio-Pharmaceutical Resources of National Education Ministry of China, Guizhou University, Guiyang, Guizhou Province 550025, China

**Keywords:** Asexual morph, new species, phylogeny, pteridophytes, taxonomy

## Abstract

During an ongoing investigation of fungi associated with ferns in southwestern China, three hyphomycetes were discovered on the dead rachises of *Angiopterisfokiensis* and an unidentified fern. Based on morphology and multi-gene phylogenetic analyses, *Arthrobotrysangiopteridis* and *Corynesporaseptata* are introduced as new species. *Arthrobotrysangiopteridis* is a nematode-trapping fungus characterized by macronematous, mononematous, hyaline conidiophores, conidiogenous cells with polyblastic denticles at each node, and 0–1-septate, clavate to elongate pyriform, hyaline conidia. *Corynesporaseptata* features macronematous, mononematous, pale brown to dark brown conidiophores, integrated, monotretic conidiogenous cells and up to 7-distoseptate with one true septum, subcylindrical to obclavate, hyaline to pale brown conidia. Detailed descriptions and illustrations of these two new species are provided, along with morphological comparisons of the new taxa with closely related species.

## ﻿Introduction

Fungi associated with ferns have historically been overlooked and have received insufficient research attention, despite being an immensely promising and diverse group (Kirschner and Liu 2014; Kirschner et al. 2019; Medel-Ortiz et al. 2020). Recent studies have provided evidence supporting this perspective, with many new species of fern-associated fungi being discovered (Guatimosim et al. 2016a; Guatimosim et al. 2016b; Li et al. 2023a; Wu and Diao 2023; Zhang et al. 2023b). China has the highest fern species, with about 2,130 species, accounting for 19% of the total global fern and fern-allies species (Lin et al. 2013; Zhou et al. 2016). Notably, the Yunnan region houses approximately 1,500 fern species, while Guizhou has about 800, ranking them as the first and third most diverse regions for fern species in China (Li et al. 2015; Zhou et al. 2016). These regions boast abundant and diverse fern resources, offering great potential for the discovery of even more interesting fungi (Zhang et al. 2023b; Hyde et al. 2024a; Phookamsak et al. 2024).

*Arthrobotrys* was introduced by Corda (1839), with *A.superba* Corda as the type species and belongs in Orbiliaceae, Orbiliales, Orbiliomycetes (Wijayawardene et al. 2022; Hyde et al. 2024b). *Arthrobotrys* is characterized by simple or branched conidiophores and obovoid, elliptic, pyriform, 0–3-septate conidia growing asynchronously on the nodes or short denticles of conidiophores (Yu et al. 2014; Zhang et al. 2022a, b, 2023a, 2024; Yang et al. 2023a; Jin et al. 2024). *Arthrobotrys* is the most complex and largest genus among Orbiliaceae nematode-trapping fungi, comprising 78 accepted species characterized by producing adhesive networks to capture nematodes (Li et al. 2005; Yang et al. 2012; Yu et al. 2014; Zhang and Hyde 2014; Jiang et al. 2017; Zhang et al. 2023a, 2024; Thiyagaraja et al. 2024). These fungal species mainly occur in soil or sediment in various ecosystems such as farmland, forests, mangroves, and freshwater. They have also been recorded in hot springs, animal waste, and tree trunks worldwide (Duddington 1954; Swe et al. 2008; Kim et al. 2001; Kumar et al. 2011; Yu et al. 2014; Jiang et al. 2017; Zhang et al. 2022a, b).

*Corynespora* was established by Güssow (1905) with *C.mazei* Güssow as the type species. *Corynespora* was placed in Corynesporascaceae as the asexual morph associated with *Corynesporasca*, based on cultural studies (Sivanesan 1996), although the latter is still accepted as a distinct genus (Wijayawardene et al. 2022; Hyde et al. 2024b; Pem et al. 2024). Phylogenetic analyses demonstrated that *Corynespora* belongs to Corynesporascaceae, Pleosporales (Voglmayr and Jaklitsch 2017; Hyde et al. 2024b; Thiyagaraja et al. 2024). The genus is characterized by distinct conidiophores; integrated, terminal, monotretic, determinate, or percurrently extending conidiogenous cells; and acrogenous, solitary or catenate, distoseptate conidia (Sharma and Chaudhary 2002; Pal et al. 2007; Voglmayr and Jaklitsch 2017; Capital and Lao 2020; Xu et al. 2020; Pem et al. 2024). Synopses of *Corynespora* species have been provided by Siboe et al. (1999), Kumar et al. (2021) and Xu et al. (2020). Subsequently, Liu et al. (2023) provided the latest list of identified and accepted species of *Corynespora* with major morphological features, host information, and locality data. *Corynespora* species have a wide distribution and can be found as saprobes, pathogens, and endophytes on living leaves, or from decaying woody material of various plants, as well as on other fungi, nematodes, and human skin (Furukawa et al. 2008; Dixon et al. 2009; Kumar and Singh 2016; Capital and Lao 2020; Xu et al. 2020; Liu et al. 2022; Liu et al. 2023). Li et al. (2023b) then introduced a new species, on branches of *Idesiapolycarpa* from Sichuan Province, China. A total of 213 epithets were listed under *Corynespora* (http://www.indexfungorum.org, accessed 20, September 2024), with 129 species being accepted (Liu et al. 2023).

In this study, collections representing two new species (*Arthrobotrysangiopteridis* and *Corynesporaseptata*) associated with ferns were made in Yunnan and Guizhou provinces in southwestern China. The identification and establishment of these taxa were based on morphological characteristics and phylogenetic evidence, a polyphasic approach, following the guidelines of Maharachchikumbura et al. (2021).

## ﻿Material and methods

### ﻿Collections, isolation and conservation

Samples of dead fern tissues were collected from Yunnan and Guizhou Provinces, China. The samples were packed in plastic bags for transportation to the laboratory, and subsequently examined using the methods described in Senanayake et al. (2020). A stereomicroscope (Leica EZ4 Microsystems (Schweiz) AG, Singapore) was used to examine and observe fungal colonies on the host surface. Morphological characteristics were documented using a Nikon DS-Ri2 digital camera fitted to a Nikon ECLIPSE Ni compound microscope (Nikon, Japan). Measurements of fungal structure were made using the Tarosoft (R) Image Frame Work, and the images used for figures were processed and combined in Adobe Illustrator CS6 (Adobe Systems, San Jose, CA, USA). Single spores were isolated following the method described by Chomnunti et al. (2014) to obtain pure cultures. Dried specimens were deposited in the Herbarium of Cryptogams, Kunming Institute of Botany, Academia Sinica (**HKAS**), Kunming, China, and the Herbarium of Guizhou Academy of Agricultural Sciences (**GZAAS**), Guiyang, China. Pure cultures were deposited in Kunming Institute of Botany Culture Collection (**KUNCC**), Kunming, China, and Guizhou Culture Collection, China (**GZCC**). Index Fungorum numbers (https://www.indexfungorum.org/Names/Names.asp) and Facesoffungi numbers (Jayasiri et al. 2015) are provided.

### ﻿DNA Extraction, PCR amplification and sequencing

Fresh fungal mycelia were scraped from the surface of colonies grown on PDA, which had been incubated at 25 °C–28 °C for one month. Fungal genomic DNA was then extracted using the Biospin Fungus Genomic DNA Extraction Kit (BioFlux®, Shanghai, China). Four partial gene regions, the nuclear ribosomal internal transcribed spacer region (ITS: ITS1-5.8S-ITS2), the partial nuclear ribosomal large subunit rRNA gene (LSU) and the partial second‐largest subunit of the RNA polymerase II gene (*rpb2*), were amplified using polymerase chain reaction (PCR). The primers used were ITS5/ITS4 for ITS (White et al. 1990), LR0R/LR5 for LSU (Vilgalys and Hester 1990) and fRPB2-5F/fRPB2-7cR for rpb2 (Liu et al. 1999). The quality of the PCR products was checked on 1% agarose electrophoresis gels stained with ethidium bromide. Purification and sequencing of PCR products were performed by Beijing Qingke Biotechnology Co., Ltd.

### ﻿Phylogenetic analyses

Original sequences were checked using BioEdit v. 7.1.3.0 (Hall 1999) and assembled using SeqMan v. 7.0.0 (DNASTAR, Madison, WI, USA). The newly assembled sequences were subjected to BLAST searches in NCBI-GenBank to preliminarily determine related taxa. The sequence data obtained from the BLAST search results and the latest publications (Li et al. 2023b; Liu et al. 2023; Jin et al. 2024; Zhang et al. 2024) were used for phylogenetic analyses. Alignments for sequences of each locus were performed with the online multiple alignment program MAFFT version 7 (https://mafft.cbrc.jp/alignment/server/, accessed on September 2024; Katoh et al. 2019), and the alignment files were further trimmed in trimAl version 1.2 (Capella-Gutiérrez et al. 2009) with the option “-gt 0.6”. Multi-gene alignments were combined using Sequence Matrix 1.7.8 (Vaidya et al. 2011). Sequences generated in this study were deposited in GenBank (Table 1 and Table 2).

**Table 1.** Taxa used in the phylogenetic analyses for *Arthrobotrys* genus, and their GenBank accession numbers.

Taxa	Strain Number	ITS	*tef1-α*	*rpb2*
*Arthrobotrysamerospora*	CBS 268.83	NR_159625	N/A	N/A
*Arthrobotrysangiopteridis*	KUNCC 23-14121	PQ346307	N/A	PQ356383
*Arthrobotrysangiopteridis*	KUNCC 23-14119	PQ346306	N/A	N/A
*Arthrobotrysanomala*	YNWS02-5-1	AY773451	AY773393	AY773422
*Arthrobotrysarthrobotryoides*	AOAC	MF926580	N/A	N/A
*Arthrobotrysblastospora*	CGMCC 3.20940	OQ332405	OQ341651	OQ341649
*Arthrobotrysbotryospora*	CBS 321.83	NR_159626	N/A	N/A
*Arthrobotryscibiensis*	DLUCC 109	OR880379	OR882792	OR882797
*Arthrobotryscibiensis*	EY10	OR902195	OR882787	OR882802
*Arthrobotryscladodes*	1.03514	MH179793	MH179616	MH179893
*Arthrobotrysclavispora*	CBS 545.63	MH858353	N/A	N/A
*Arthrobotrysconoides*	670	AY773455	AY773397	AY773426
*Arthrobotryscookedickinson*	YMF 1.00024	MF948393	MF948550	MF948474
*Arthrobotryscystosporia*	CBS 439.54	MH857384	N/A	N/A
*Arthrobotrysdendroides*	YMF 1.00010	MF948388	MF948545	MF948469
*Arthrobotrysdianchiensis*	1.00571	MH179720	N/A	MH179826
*Arthrobotryselegans*	1.00027	MH179688	N/A	MH179797
*Arthrobotryseryuanensis*	CGMCC 3.19715	MT612105	OM850307	OM850301
*Arthrobotryseudermata*	SDT24	AY773465	AY773407	AY773436
*Arthrobotrysflagrans*	1.01471	MH179741	MH179583	MH179845
*Arthrobotrysgampsospora*	CBS 127.83	U51960	N/A	N/A
*Arthrobotrysglobospora*	1.00537	MH179706	MH179562	MH179814
*Arthrobotrysgongshanensis*	CGMCC 3.23753	OM801277	OM809162	OM809163
*Arthrobotrysguizhouensis*	YMF 1.00014	MF948390	MF948547	MF948471
*Arthrobotrysheihuiensis*	DLUCC 108-1	OR880378	OR882791	OR882796
*Arthrobotrysheihuiensis*	Y710	OR902194	OR882786	OR882801
*Arthrobotryshengjiangensis*	CGMCC 3.24983	OQ946587	OQ989312	OQ989302
*Arthrobotryshyrcanus*	IRAN 3650C	MH367058	OP351540	N/A
*Arthrobotrysindica*	YMF 1.01845	KT932086	N/A	N/A
*Arthrobotrysiridis*	521	AY773452	AY773394	AY773423
*Arthrobotrysjanus*	Jan-85	AY773459	AY773401	AY773430
*Arthrobotrysjavanica*	105	EU977514	N/A	N/A
*Arthrobotrysjindingensis*	CGMCC 3.20895	OP236810	OP272511	OP272515
*Arthrobotrysjinpingensis*	CGMCC 3.20896	OM855569	OM850311	OM850305
*Arthrobotrysjinshaensis*	DLUCC 133	OR880381	OR882794	OR882799
*Arthrobotrysjinshaensis*	MA142	OR902197	OR882789	OR882804
*Arthrobotryskoreensis*	C45	JF304780	N/A	N/A
*Arthrobotryslanpingensis*	CGMCC 3.20998	OM855566	OM850308	OM850302
*Arthrobotryslatispora*	H.B. 8952	MK493125	N/A	N/A
*Arthrobotryslongiphora*	1.00538	MH179707	N/A	MH179815
*Arthrobotryslunzhangensis*	CGMCC 3.20941	OK643973	OM621809	OM621810
*Arthrobotrysluquanensis*	CGMCC 3.20894	OM855567	OM850309	OM850303
*Arthrobotrysmangrovispora*	MGDW17	EU573354	N/A	N/A
*Arthrobotrysmegalospora*	TWF800	MN013995	N/A	N/A
*Arthrobotrysmicroscaphoides*	YMF 1.00028	MF948395	MF948552	MF948476
*Arthrobotrysmultiformis*	CBS 773.84	MH861834	N/A	N/A
*Arthrobotrysmusiformis*	SQ77-1	AY773469	AY773411	AY773440
*Arthrobotrysmusiformis*	1.03481	MH179783	MH179607	MH179883
*Arthrobotrysnonseptata*	YMF 1.01852	FJ185261	N/A	N/A
*Arthrobotrysobovata*	YMF 1.00011	MF948389	MF948546	MF948470
*Arthrobotrysoligospora*	920	AY773462	AY773404	AY773433
*Arthrobotryspaucispora*	ATCC 96704	EF445991	N/A	N/A
*Arthrobotryspolycephala*	1.01888	MH179760	MH179592	MH179862
*Arthrobotryspseudoclavata*	1130	AY773446	AY773388	AY773417
*Arthrobotryspsychrophila*	1.01412	MH179727	MH179578	MH179832
*Arthrobotryspyriformis*	YNWS02-3-1	AY773450	AY773392	AY773421
*Arthrobotrysreticulata*	CBS 550.63	MH858355	N/A	N/A
*Arthrobotrysrobusta*	nefuA4	MZ326655	N/A	N/A
*Arthrobotryssalina*	SF 0459	KP036623	N/A	N/A
*Arthrobotrysscaphoides*	1.01442	MH179732	MH179580	MH179836
*Arthrobotrysshizishanna*	YMF 1.00022	MF948392	MF948549	MF948473
*Arthrobotrysshuifuensis*	CGMCC 3.19716	MT612334	OM850306	OM850300
*Arthrobotryssinensis*	105-1	AY773445	AY773387	AY773416
*Arthrobotryssphaeroides*	1.0141	MH179726	MH179577	MH179831
*Arthrobotryssuperba*	127	EU977558	N/A	N/A
*Arthrobotrysthaumasia*	917	AY773461	AY773403	AY773432
*Arthrobotrystongdianensis*	CGMCC 3.20942	OP236809	OP272509	OP272513
*Arthrobotrysvermicola*	629	AY773454	AY773396	AY773425
*Arthrobotrysweixiensis*	CGMCC 3.24984	OQ946585	OQ989310	OQ989300
*Arthrobotrysxiangyunensis*	YXY10-1	MK537299	N/A	N/A
*Arthrobotrysyangbiensis*	DLUCC 36-1	OR880382	OR882795	OR882800
*Arthrobotrysyangbiensis*	Y678	OR902198	OR882790	OR882805
*Arthrobotrysyangjiangensis*	DLUCC 124	OR880380	OR882793	OR882798
*Arthrobotrysyangjiangensis*	YB19	OR902196	OR882788	OR882803
*Arthrobotrysyunnanensis*	YMF 1.00593	AY50993	N/A	N/A
*Arthrobotryszhaoyangensis*	CGMCC 3.20944	OM855568	OM850310	OM850304
*Dactylellinacangshanensis*	CGMCC 3.19714	MK372062	MN915115	MN915114
*Dactylellinacopepodii*	CBS 487.90	U51964	DQ999835	DQ999816

Note: “N/A” indicates no data are available in GenBank. The newly generated sequences are indicated in blue.

**Table 2.** Taxa used in the phylogenetic analyses for *Corynespora* genus, and their GenBank accession numbers.

Taxa	Strain Number	ITS	LSU
*Corynesporacassiicola*	CBS 100822	N/A	GU301808
*Corynesporacitricola*	CBS 169.77	FJ852594	N/A
*Corynesporadoipuiensis*	MFLUCC 14-0022	MN648322	MN648326
*Corynesporaencephalarti*	CBS 145555	MK876383	MK876424
*Corynesporalignicola*	MFLUCC 16–1301	MN860549	MN860554
*Corynesporamengsongensis*	HJAUP C2000T	OQ060574	OQ060578
*Corynesporanabanheensis*	HJAUP C2048T	OQ060577	OQ060580
*Corynesporapseudocassiicola*	CPC 31708	MH327794	MH327830
*Corynesporaseptata*	GZCC 23-0741	PQ346308	PQ346311
*Corynesporasmithii*	L120	KY984297	KY984297
*Corynesporasmithii*	L130	KY984298	KY984298
*Corynesporasmithii*	CABI 5649b	FJ852597	GU323201
*Corynesporasmithii*	CBS 139925	KY984299	KY984299
*Corynesporasubmersa*	MFLUCC 16–1101	MN860548	MN860553
*Corynesporatorulosa*	CBS 136419	MH866095	MH877634
*Corynesporathailandica*	CBS 145089	MK047455	MK047505
*Corynesporayunnanensis*	HJAUP C2132T	OQ060579	OQ060583
*Periconiabyssoides*	H 4600	LC014581	AB807570
*Periconiadigitata*	CBS 510.77	LC014584	AB807561
*Periconiapseudodigitata*	KT 1395	NR_153490	NG_059396
*Periconiapseudodigitata*	UESTCC 23.0022	OR253146	OR253305
*Periconiapseudodigitata*	UESTCC 23.0023	OR253147	OR253306

Note: “N/A” indicates no data are available in GenBank. The newly generated sequences are indicated in blue.

The fasta files were converted to the formats required for the AliView program (Larsson 2014), PHYLIP for maximum likelihood analysis (ML), and NEXUS for Bayesian analysis (BYPP). Maximum likelihood (ML) analyses were performed using RAxML-HPC Blackbox (8.2.10) tool on the XSEDE Teragrid at the CIPRES Science Gateway (https://www.phylo.org; accessed on 10 September 2024), with rapid bootstrap analysis followed by 1,000 bootstrap replicates (Miller et al. 2010; Stamatakis 2014). The final tree was selected from the suboptimal trees of each run by comparing likelihood scores under the GTRGAMMA substitution model. Bayesian analyses were performed by MrBayes 3.2.7a on XSED via CIPRES (Miller et al. 2010). MrModeltest v.2.3 was used to determine the best nucleotide substitution model for each data partition (Nylander 2004). Posterior probabilities (PP) (Rannala and Yang 1996) were calculated using the Bayesian Markov Chain Monte Carlo (BMCMC) sampling method (Huelsenbeck 2001; Zhaxybayeva and Gogarten 2002). Four simultaneous Markov chains were run for 1 million generations, with trees sampled every 100^th^ generations, yielding 10,000 trees. Phylogenetic trees were visualized using FigTree v. 1.4.4 (Rambaut 2014), and the layouts were created using Adobe Illustrator CS5 software (Adobe Systems, San Jose, CA, USA). The newly obtained sequences in this study were deposited in GenBank.

## ﻿Taxonomy

### ﻿ Arthrobotrys angiopteridis

Taxon classificationFungiOrbilialesOrbiliaceae

J.Y. Zhang, Y.Z. Lu & K.D. Hyde sp. nov.

3178D694-C678-5DDA-948E-B4049C0CF457

902682

Facesoffungi number: FoF16618

Fig. 2

#### Etymology.

Named after the fungal host genus *Angiopteris*.

#### Holotype.

HKAS 129855.

#### Description.

***Saprobic*** on dead rachis of *Angiopterisfokiensis* in terrestrial habitats. **Sexual morph** Undetermined. **Asexual morph *Colonies*** on natural substrate superficial, effuse, hyaline, with white and glistening masses of conidia on the apex of conidiophores. ***Mycelium*** partly superficial, partly immersed, composed of septate, branched, smooth hyphae. ***Conidiophores*** 345–502 µm long, 6–8.5 µm wide at the base (*x̄* = 418 × 6.9 µm, n = 20), macronematous, mononematous, solitary, erect, straight or slightly flexuous, unbranched, cylindrical, septate, smooth-walled, hyaline. ***Conidiogenous cells*** 95–176 × 2–4.5 µm (*x̄* = 129 × 3.5 µm, n = 20), polyblastic, producing 1–5 separate nodes by the repeated elongation, with multi polyblastic denticles at each node, hyaline. ***Conidia*** 25–35 × 8–11 µm (*x̄* = 28.8 × 9 µm, n = 25), aseptate, or 1-septate at the median to submedian, not constricted or slightly constricted at the septum, clavate to elongate pyriform, broadly rounded at apex, pointed or sometimes truncate at the base, sometimes with a bud-like projection at base, straight or slightly curved, smooth-walled or rough walled, guttulate, hyaline.

#### Culture characteristics.

Conidia germinating on WA within 15 h and germ tube produced from conidia. ***Colonies*** growing on PDA, reaching 60 mm diameter in 10 days at 26 °C, circular, cottony, white, and not producing pigmentation in culture.

#### Material examined.

China • Guizhou Province, Zunyi City, Xishui County (28°22'19"N, 106°0'35"E), on dead rachis of *Angiopterisfokiensis* (Marattiaceae) in a disturbed forest nearby the roadside, 13 April 2023, J.Y. Zhang, ZY06 (HKAS 129855, holotype; GZAAS 23–0758, isotype), ex-type living culture, KUNCC 23–14121; • ibid., ZY02 (HKAS 129854, paratype), ex-paratype living culture, KUNCC 23–14119. Additional sequence: KUNCC 23–14121: PQ346313 (SSU) and PQ346310 (LSU); KUNCC 23–14119: PQ346312 (SSU) and PQ346309 (LSU).

#### Notes.

**Figure 1.**
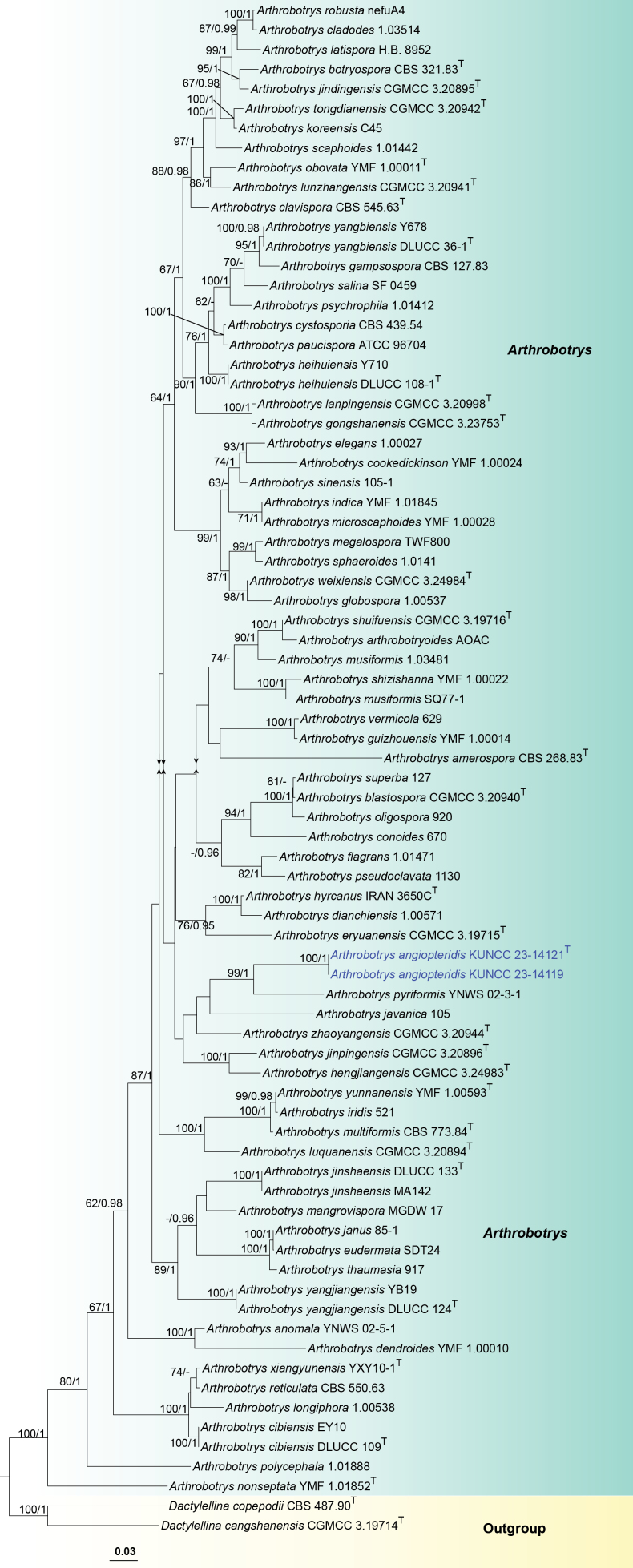
Phylogram generated from maximum likelihood analysis based on combined ITS, *tef1-α* and *rpb2* sequence data. Seventy-eight taxa were included in the combined analyses, which comprised 1920 characters (ITS = 583 bp, *tef1-α* = 512 bp and *rpb2* = 825 bp) after alignment. The best scoring RAxML tree with a final likelihood value of -22800.405782 is presented. The matrix had 983 distinct alignment patterns, with 25.05% of undetermined characters or gaps. Estimated base frequencies were as follows: A = 0.260557, C = 0.263561, G = 0.230377, T = 0.245504; substitution rates: AC = 1.414531, AG = 3.978691, AT = 1.319991, CG = 0.945884, CT = 6.618473, GT = 1.000000; gamma distribution shape parameter *α* = 0.262034. Bootstrap support values for ML equal to or greater than 60% and prior probabilities (PPs) equal to or greater than 0.95 are given above the nodes as ML/PP. The tree was rooted to *Dactylellinacopepodii* (CBS 487.90) and *D.cangshanensis* (CGMCC 3.19714). The strain numbers are noted after the species names with ex-type strains indicated by ^T^. The newly generated sequences are indicated in blue.

Phylogenetically, the new isolates KUNCC 23–14121 and KUNCC 23–14119 of *Arthrobotrysangiopteridis* clustered together formed a separate clade with 100% ML/1.00 PP bootstrap support and are sister to *A.pyriformis* (Fig. 1). A comparison of nucleotide base pairs between them reveals differences of 30/459 (6.5%, including 15 gaps) and 82/730 bp (11%, no gap) in the ITS and *rpb2* sequences, respectively. This indicates that they are distinct species. Morphologically, *A.angiopteridis* aligns well with the generic concept and resembles *A.oligospora* in having hyaline conidiophores with the successive production of additional denticle nodes (Yu et al. 2014). However, *A.angiopteridis* can be easily distinguished from *A.oligospora* by its longer conidiophores (345–502 µm vs. 110–440 μm) and clavate to elongate pyriform conidia, with 0–1 septate near the middle, whereas *A.oligospora* has pyriform or obovoid conidia with 1-septate near the base. Therefore, we introduce *A.angiopteridis* as a novel species based on its distinct morphological and phylogenetic evidence following the guidelines of Maharachchikumbura et al. (2021).

### ﻿ Corynespora septata

Taxon classificationFungiPleosporalesCorynesporascaceae

J.Y. Zhang, Y.Z. Lu & K.D. Hyde sp. nov.

D48F6871-0287-5833-84C4-B3A50209A290

902683

Facesoffungi number: FoF16619

Fig. 4

#### Etymology.

Named after the presence of eu-septate conidia.

#### Holotype.

HKAS 129839.

#### Description.

***Saprobic*** on dead rachis of an unidentified fern in terrestrial habitats. **Sexual morph** undetermined. **Asexual morph *Colonies*** on natural substrate superficial, effuse, gregarious, hairy, brown to black. ***Mycelium*** partly superficial, partly immersed, composed of branched, septate, pale brown to brown, smooth-walled hyphae. ***Conidiophores*** 490–671 µm long, 3.5–6.5 µm wide at the base (*x̄* = 600 × 5 µm, n = 15), macronematous, mononematous, erect, straight or flexible, unbranched, or occasionally branched, septate, smooth, dark brown at the base, pale towards the apex. ***Conidiogenous cells*** 21–60 × 3–5.5 µm (*x̄* = 36.3 × 3.8 µm, n = 15), integrated, terminal, monotretic, cylindrical, smooth, pale brown to brown. ***Conidia*** 42–74 × 4.5–7.5 µm (*x̄* = 54 × 5.7 µm, n = 25), acrogenous, solitary, up to 7-distoseptate with one true septum, straight or slightly curved, subcylindrical to obclavate, rounded at the apex, base short obconically truncate, somewhat thickened and darkened, sometimes with percurrent proliferation which forms another conidium from the conidial apex, hyaline to pale brown.

#### Culture characteristics.

Conidia germinating on WA within 15 h and germ tube produced from conidia. ***Colonies*** growing on PDA, reaching 55 mm diameter in 10 days at 26 °C, circular, flat with entire margin, velvety, fluffy, white from above, reverse dark brown at center, paler to light yellow towards margin, and not producing pigmentation in culture.

#### Material examined.

China • Yunnan Province, Xishuangbanna Dai Autonomous Prefecture, Mengla County, Menglun Town, Xishuangbanna Tropical Botanical Garden, Chinese Academy of Sciences (21°55'39"N, 101°15'15"E), on dead rachis of an unidentified fern, 16 November 2019, J.Y. Zhang, Y159 (HKAS 129839, holotype; GZAAS 23–0769, isotype), ex-type living culture, GZCC 23–0741.

#### Notes.

A BLASTn search in NCBI-GenBank revealed that the LSU and ITS sequences of our newly collected strain of *Corynesporaseptata* exhibited 99% similarity to *C.encephalarti* (NG_067878) and 95.62% similarity to *C.cassiicola* (MN648322), respectively. Phylogenetic analysis confirmed that *C.septata* formed a distinct clade within *Corynespora* and shared a sister relationship with *C.pseudocassiicola* Crous & M.J. Wingf. (Fig. 3). There are 10 bp (10/841 bp with 0 gap, 1%) and 38 bp (38/527 bp with 13 gaps, 7%) differences between the *C.pseudocassiicola* and *C.septata* in the LSU and ITS gene regions, respectively. Morphologically, *C.septata* has longer conidiophores (490–671 µm vs. 200–400 µm), and smaller conidia (42–74 × 4.5–7.5 µm vs. 95–160 × 9–10 µm) compared to *C.pseudocassiicola* (Crous et al. 2018). Similarly, *C.septata* is most similar to *C.lignicola* Z.L. Luo, H.Y. Su & K.D. Hyde in the shapes of conidiophores, conidiogenous cells, and conidia (Capital and Lao 2020). However, *C.septata* differs from *C.lignicola* in having narrower conidiophores (3.5–6.5 µm vs. 9–13 µm) and notably smaller conidia (42–74 × 4.5–7.5 µm vs. 110–156 × 7–9 µm).

## ﻿Discussion

During a survey of bracken (*Pteridiurnaquilinum* (L.) Kuhn) petiole decomposition in the United Kingdom, *Arthrobotrysmegalosporus* (Drechsler) M. Scholler, Hagedorn & A. Rubner (Synonym: *Dactylellamegalospora* Drechsler) was found to be a member of the common fungi (Frankland 1976). This is also the only record of *Arthrobotrys* species being associated with ferns. *Corynesporacassiicola* (Berk. & M.A. Curtis) C.T. Wei and an unidentified *Corynespora* species have been discovered on ferns in United States (Alfieri et al. 1984; Smith 2008; Farr et al. 2021). Specifically, *C.cassiicola* has been found on six fern species including *Arachniodesaristata* (Davalliaceae), *Athyriumniponicum* (Dryopteridaceae), *Adiantumcuneatum* (Adiantaceae), *Adiantumtenerum* (Adiantaceae), *Davalliarepens* (Davalliaceae), and *Platycerium* spp. (Pteridaceae) (Alfieri et al. 1984; Smith 2008). Additionally, an unidentified *Corynespora* species was collected from *Nephrolepisexaltata* (Davalliaceae) (Alfieri et al. 1984; Farr et al. 2021). Based on phylogenetic and morphological evidence, *Arthrobotrysangiopteridis* and *Corynesporaseptata*, isolated from ferns are reported as new species in this study from Yunnan and Guizhou provinces. These findings contribute to a better understanding of fern-related fungi and aim to enhance attention and awareness of fungal communities associated with ferns.

Most *Corynespora* species were introduced based on morphology (Sivanesan 1996; Siboe et al. 1999; Sharma and Chaudhary 2002; Kumar and Singh 2016; Liu et al. 2023). However, distinguishing between *Corynespora* and some similar genera, especially *Helminthosporium*, based solely on morphology has proven to be challenging (Castañeda Ruiz et al. 2004; Voglmayr and Jaklitsch 2017). The application of molecular data has confirmed this difficulty. For example, *Corynesporacaespitosa*, *C.endiandrae*, *C.leucadendri* and *C.olivacea* were transferred to *Helminthosporium* based on phylogenetic evidence (Voglmayr and Jaklitsch 2017). Currently, sequence data is available in GenBank for only 15 species. Therefore, it is essential to obtain more collections with sequence data for verification of *Corynespora* species.

**Figure 2.**
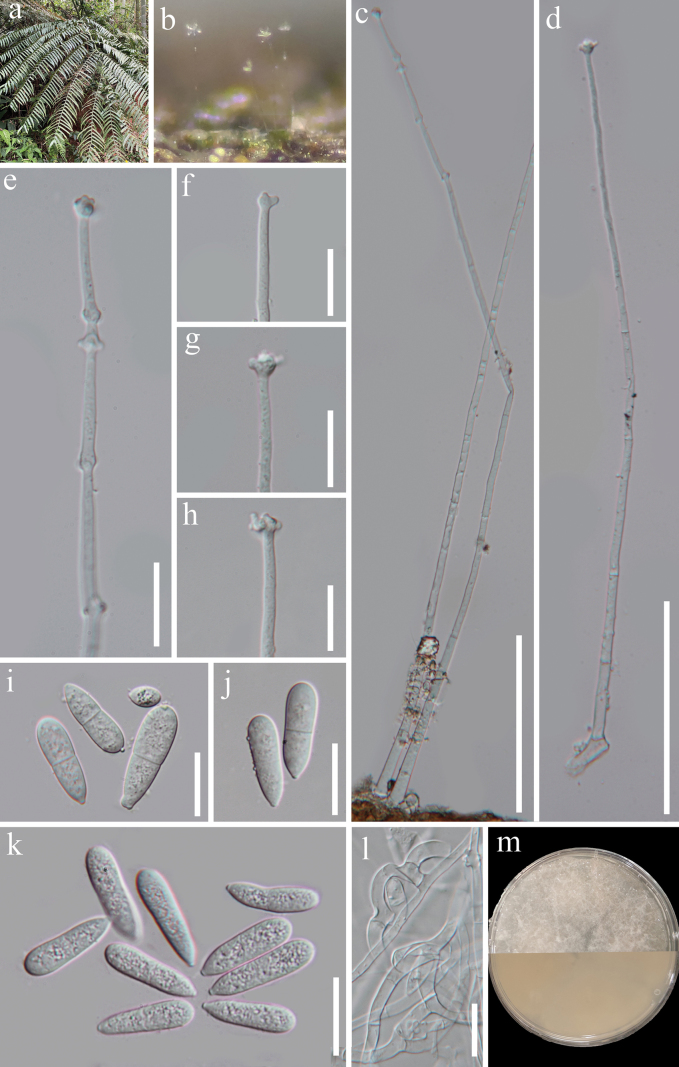
*Arthrobotrysangiopteridis* (HKAS 129855, holotype) **a** the host **b** colonies on the host **c, d** conidiophores with conidiogenous cells **e–h** conidiogenous cells **i–k** conidia **l** trapping mycelia: adhesive networks **m** pure culture from front and reverse. Scale bar: 100 µm (**c, d**); 20 µm (**e–l**).

*Arthrobotrysangiopteridis* sp. nov., isolated from *Angiopterisfokiensis*, is a member of nematode-trapping fungi with trapping device of adhesive networks (Fig. 2l). Nematode-trapping fungi are crucial for preserving ecological balance and possess the potential for biologically controlling harmful nematodes (Jiang et al. 2017; Zhang et al. 2024). *Arthrobotrysangiopteridis* is a valuable fungus that is expected to contribute to the exploration of ecological protection in the future.

**Figure 3.**
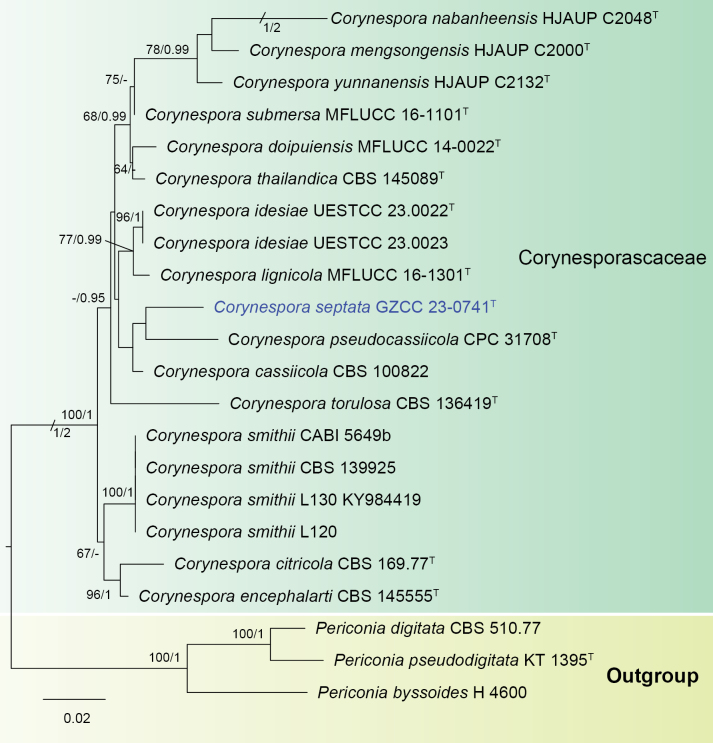
Phylogram generated from maximum likelihood analysis based on combined LSU and ITS sequence data. Twenty-two taxa were included in the combined analyses, which comprised 1393 characters (LSU = 844 bp and ITS = 549 bp) after alignment. The best scoring RAxML tree with a final likelihood value of -4677.993509 is presented. The matrix had 336 distinct alignment patterns, with 9.65% of undetermined characters or gaps. Estimated base frequencies were as follows: A = 0.243499, C = 0.247400, G = 0.289966, T = 0.219136; substitution rates: AC = 3.067252, AG = 2.397685, AT = 1.551875, CG = 1.069828, CT = 6.624253, GT = 1.000000; gamma distribution shape parameter *α* = 0.240919. Bootstrap support values for ML equal to or greater than 60% and prior probabilities (PPs) equal to or greater than 0.95 are given above the nodes as ML/PP. The tree was rooted to *Periconiabyssoides* (H 4600), *P.digitata* (CBS 510.77) and *P.pseudodigitata* (KT 1395). The strain numbers are noted after the species names with ex-type strains indicated by ^T^. The newly generated sequences are indicated in blue.

**Figure 4.**
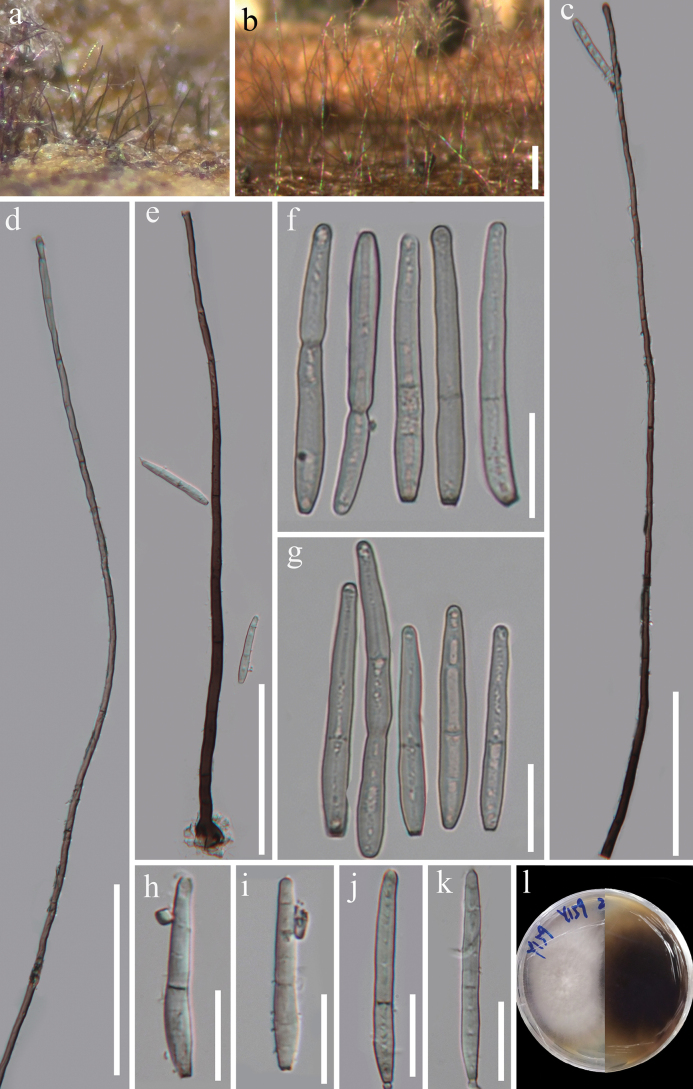
*Corynesporaseptata* (HKAS 129839, holotype) **a, b** colonies on the host **c–e** conidiophores with conidiogenous cells **f–k** conidia **l** pure culture from front and reverse. Scale bar: 200 µm (**b**); 100 µm (**c–e**); 20 µm (**f–k**).

Yunnan and Guizhou provinces are not only the most abundant areas for fern plants in China (Li et al. 2015; Zhou et al. 2016), but also hotspots for the discovery of new fungal species (Wang et al. 2021; Yang et al. 2023b; Zhang et al. 2023b; Dissanayake et al. 2024). The introduction of *A.angiopteridis* and *C.septata* adds to the growing evidence of high fungal diversity in Guizhou and Yunnan province, China (Dissanayake et al. 2024; Dong et al. 2024; Li et al. 2024).

## Supplementary Material

XML Treatment for Arthrobotrys angiopteridis

XML Treatment for Corynespora septata

## ﻿Additional information

### Author ORCIDs

Jing-Yi Zhang https://orcid.org/0000-0003-0606-6169

Kevin D. Hyde https://orcid.org/0000-0002-2191-0762

Li-Juan Zhang https://orcid.org/0000-0002-3234-6757

Song Bai https://orcid.org/0000-0002-1972-2834

Dan-Feng Bao https://orcid.org/0000-0002-5697-4280

Fatimah Al-Otibi https://orcid.org/0000-0003-3629-5755

Yong-Zhong Lu https://orcid.org/0000-0002-1033-5782

### Data availability

All of the data that support the findings of this study are available in the main text.
